# Effects of 3-(4-Hydroxy-3-methoxyphenyl)propionic Acid on Enhancing Grip Strength and Inhibiting Protein Catabolism Induced by Exhaustive Exercise

**DOI:** 10.3390/ijms25126627

**Published:** 2024-06-16

**Authors:** Yishan Tong, Jiapeng Huang, Shuo Wang, Riyo Awa, Takashi Tagawa, Ziwei Zhang, Tiehan Cao, Haruki Kobori, Katsuhiko Suzuki

**Affiliations:** 1 Graduate School of Sport Sciences, Waseda University, Tokorozawa 359-1192, Japan; tongyishan130@ruri.waseda.jp (Y.T.); hjpshidsg1234@toki.waseda.jp (J.H.); wang_sh@akane.waseda.jp (S.W.); zhangziwei@fuji.waseda.jp (Z.Z.); caotiehan0313@toki.waseda.jp (T.C.); koboharu1223@fuji.waseda.jp (H.K.); 2 Research Center, Maruzen Pharmaceuticals Co., Ltd., Fukuyama, Hiroshima 729-3102, Japan; r-awa@maruzenpcy.co.jp (R.A.); t-tagawa@maruzenpcy.co.jp (T.T.); 3 Faculty of Sport Sciences, Waseda University, Tokorozawa 359-1192, Japan

**Keywords:** 3-(4-hydroxy-3-methoxyphenyl)propionic acid, grip strength, exhaustive exercise, glucose metabolism, lipid metabolism, protein catabolism

## Abstract

3-(4-Hydroxy-3-methoxyphenyl)propionic acid (HMPA), also known as dihydroferulic acid, is a hydroxycinnamic acid derivative that can be derived from the microbial transformation of dietary polyphenols or naturally obtained from fermented foods. Although numerous studies have documented its antioxidant and anti-obesity effects, the effect of HMPA on muscle function remains unknown. This study investigated the effects of HMPA on muscle strength and exercise endurance capacity. Mice were orally administered low and high doses of HMPA for 14 days and subjected to grip force and treadmill exhaustion tests to evaluate muscle function. Our results showed that HMPA-administered groups significantly enhanced absolute grip strength (*p* = 0.0256) and relative grip strength (*p* = 0.0209), and low-dose HMPA decreased the plasma level of blood urea nitrogen after exercise (*p* = 0.0183), but HMPA did not affect endurance performance. Low-dose HMPA administration increased *Myf5* expression in sedentary mice (*p* = 0.0106), suggesting that low-dose HMPA may promote muscle development. Additionally, HMPA improved hepatic glucose and lipid metabolism, and inhibited muscular lipid metabolism and protein catabolism, as indicated by changes in mRNA expression levels of related genes. These findings suggest that HMPA may be a promising dietary supplement for muscle health and performance.

## 1. Introduction

Skeletal muscle, the most abundant tissue in the body, plays a crucial role in various biological activities, such as physical movement, thermogenesis, and energy metabolism [1,2]. It functions as a major site for glycogenesis and as a protein reservoir to maintain energy and protein balance [2]. Aging, sedentary lifestyles, and unhealthy diets have detrimental effects on skeletal muscle functionality, leading to muscle dysfunction [3,4].

The improvement of muscle function is of great interest to varied populations, not only to people with skeletal muscle dysfunction but also to athletes driven by competitive motivations [5]. The improvement of muscle function is governed by the combined action of many regulatory factors, such as improving metabolism and oxidative capacity in various tissues [6]. Additionally, improving the expression of myogenic regulatory factors (MRFs) and muscle protein synthesis is crucial for muscle regeneration and increased muscle mass [5,7]. More importantly, those improvements can offer protection under various pathological conditions, including inflammation, ischemia, and hypoxia [8]. Recent studies have increasingly reported that phenolic compounds improve muscle strength, exercise performance, and/or reduce tissue damage resulting from physical challenge [9,10].

**Figure 1:**
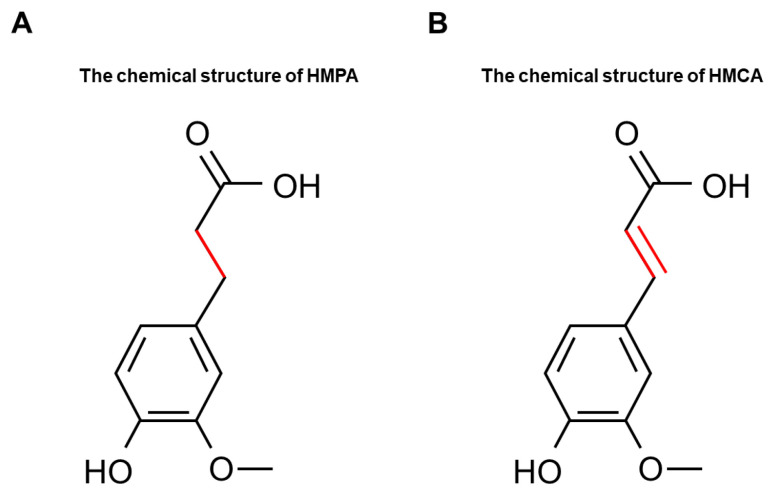
The chemical structure of (**A**) HMPA and (**B**) HMCA. HMPA, 3-(4-hydroxy-3-methoxyphenyl)propionic acid; HMCA, 4-hydroxy-3-methoxycinnamic acid.

3-(4-hydroxy-3-methoxyphenyl)propionic acid (HMPA) (Figure 1A), also known as dihydroferulic acid, is a phenolic compound that can be naturally found in various fermented foods, such as vinegar [11] and fermented rice bran [12]. It is also a microbial metabolite from the degradation of dietary polyphenols like 4-hydroxy-3-methoxycinnamic acid (HMCA) (Figure 1B), known as ferulic acid [13,14,15]. HMPA and HMCA share some chemical properties due to their similar chemical structures. However, HMPA exhibits unique characteristics as it forms through the hydrogenation of the double bond in the HMCA molecule. HMCA is a well-studied phenolic acid, known for its pharmacological properties, including antioxidant [16], anti-inflammatory [17], and hypolipidemic effects [13]. Growing research has demonstrated a variety of positive effects of HMCA on muscle function. Wang et al. [18] demonstrated that a five-week dietary supplementation with 0.45% HMCA could increase the proportion of slow-twitch fibers without affecting growth performance. Similarly, a 30-day dietary supplementation with 0.2% HMCA led to an increase in muscle mass [19]. Additionally, You et al. [20,21] showed that both a single dose (125 or 250 μmole/kg) and a 12-day oral administration of 25 mg/kg of HMCA enhanced exercise endurance capacity in mice, attributed to its antioxidant effects. However, the applications of HMCA are limited because of its low water solubility and rapid oxidation [22,23].

The derivatives of HMCA have demonstrated promising features, including superior antioxidant activity, enhanced stability, and reduced toxicity, offering a wide range of applications [15,24,25]. Several in vitro studies have shown that HMPA demonstrates superior antioxidant [15] and anti-platelet activities [25] compared to HMCA. Shimoji et al. [15] identified HMPA, present in Kurosu (unpolished rice vinegar), as exhibiting a higher level of antioxidative activity than HMCA, suggesting that HMPA may have advantageous applications in antioxidative food products due to its better water solubility. Ohue-Kitano et al. [26] observed a higher absorption rate of HMPA than HMCA following an oral administration of 500 mg/kg in mice. Moreover, in antibiotic-treated mice where HMPA production was absent, HMCA administration failed to produce anti-obesity effects or enhance hepatic lipid metabolism, indicating a critical role of HMPA in facilitating the positive metabolic effects associated with HMCA [26]. Considering the potent bioactivity of HMPA, we hypothesize that HMPA administration would improve muscle function in mice. To test this hypothesis, two doses of HMPA were applied to investigate the chronic effects of HMPA on muscle function, including muscle strength and exercise endurance capacity. To understand the underlying molecular mechanisms, we analyzed plasma biomarkers associated with tissue damage and energy substances, in addition to assessing mRNA expression related to energy metabolism and muscle development pathways.

## 2. Results

### 2.1. Effects of HMPA Administration on Skeletal Muscle Function in Mice

Two different doses of HMPA were orally administered to mice for 14 days and did not affect body weight compared to in the vehicle control group (Figure S1).

**Figure 2:**
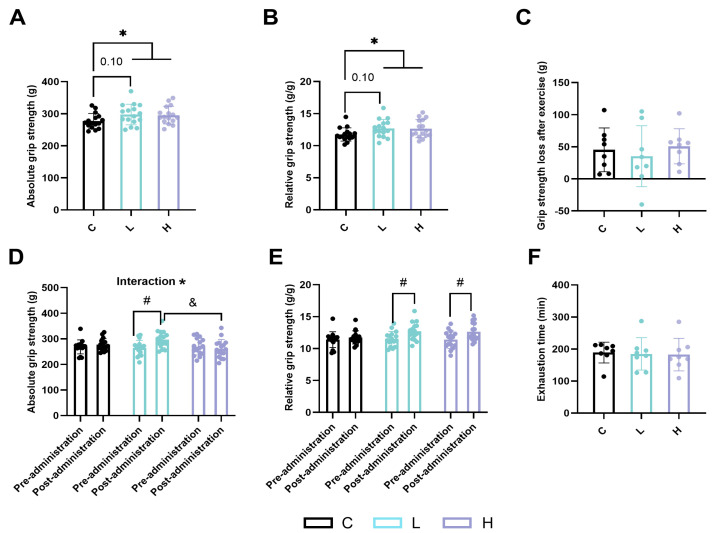
(**A**) The absolute grip strength at post-administration. (**B**) The relative grip strength at post-administration. (**C**) The loss of grip strength after exercise. (**D**) The changes in absolute grip strength before and after administration. (**E**) The changes in relative grip strength before and after administration. (**F**) The exhaustion time in exercise groups. C group, vehicle control group; L group, low-dose HMPA administration group; H group, high-dose HMPA administration group. Values are the means ± standard deviation (SD); * *p* < 0.05, compared with the C group; # *p* < 0.05, compared with pre-administration; & *p* < 0.05, compared with the H group at post-administration.

Low-dose HMPA tended to increase both relative and absolute grip strength after the administration, but the difference did not reach statistical significance (Figure 2A,B, *p* = 0.10). Compared to in the C group, both absolute and relative grip strength were significantly improved in the HMPA-administered (L + H) groups (Figure 2A,B, *p* < 0.05).

Regarding the difference in grip strength before and after administration, there was a statistically significant main effect of time in the analysis of the absolute grip strength (Figure 2D, *p* < 0.05), and a significant time × HMPA interaction in the analysis of the relative grip strength (Figure 2E, *p* < 0.05). Post hoc evaluations indicated that absolute grip strength increased in the L group, and had a greater improvement compared to in the H group (Figure 2D, p < 0.05). The L group and the H group showed significantly increased relative grip strength (Figure 2E, *p* < 0.05), whereas there was no change in grip strength observed in the C group.

Exhaustion time appeared similar across groups (Figure 2F: 189 ± 32.57 min vs. 184.8 ± 50.43 min vs. 182.6 ± 50.83 min, *p* > 0.05). Furthermore, no significant differences were observed in the loss of grip strength after exercise (Figure 2C).

### 2.2. Effects of HMPA Administration on Visceral Function and Energy Metabolism Substances

**Figure 3:**
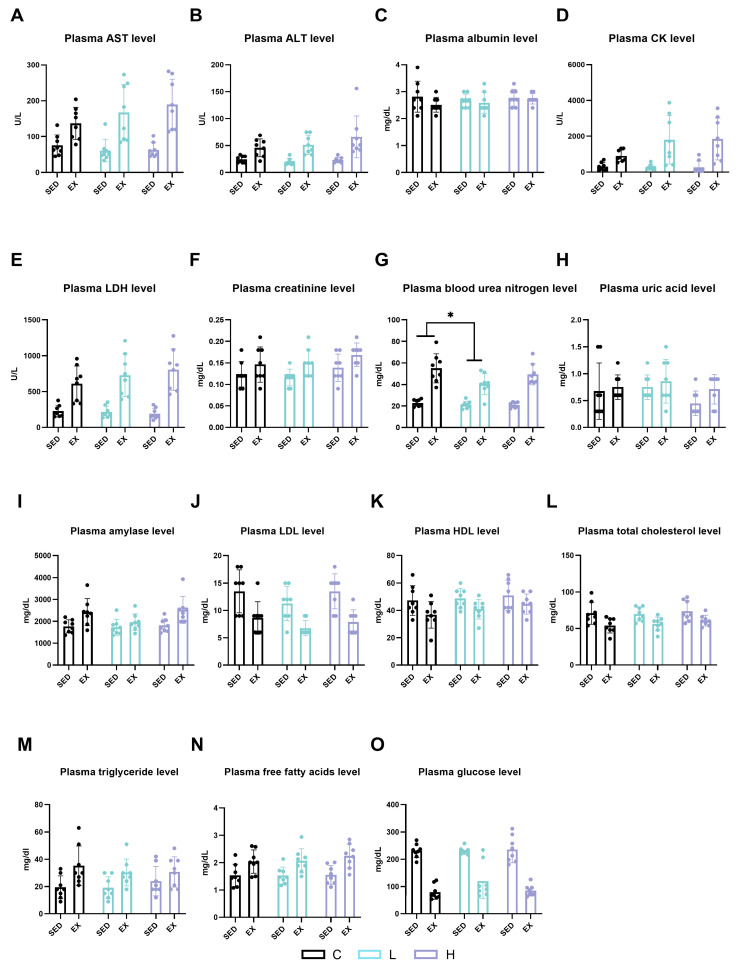
Plasma levels of (**A**) AST, (**B**) ALT, (**C**) albumin, (**D**) CK, (**E**) LDH, (**F**) creatinine, (**G**) blood urea nitrogen, (**H**) uric acid, (**I**) amylase, (**J**) LDL, (**K**) HDL, (**L**) total cholesterol, (**M**) triglyceride, (**N**) free fatty acids, and (**O**) glucose. AST, aspartate aminotransferase; ALT, alanine aminotransferase; CK, creatine kinase; LDH, lactate dehydrogenase; LDL, low-density lipoprotein cholesterol; HDL, high-density lipoprotein cholesterol; C group, vehicle control group; L group, low-dose HMPA administration group; H group, high-dose HMPA administration group; SED, sedentary; EX, exercise. Values are the means ± standard deviation (SD); * *p* < 0.05, compared with the C group.

Numerous biochemical markers in plasma were assessed to investigate the effects of a 14-day oral administration of HMPA on visceral functions and energy metabolism, as well as the effects of HMPA on exhaustive exercise. There were no significant differences among the C group, L group, and H group at post-administration in the blood markers of albumin, aspartate aminotransferase (AST), alanine aminotransferase (ALT), blood urea nitrogen (BUN), creatinine (Cr), lactate dehydrogenase (LDH), creatine kinase (CK), and amylase, demonstrating that 14 days of HMPA administration did not result in toxic effects (Figure 3). Exhaustive exercise significantly increased the plasma levels of AST (Figure 3A, *p* < 0.0001), ALT (Figure 3B, *p* < 0.0001), CK (Figure 3D, *p* < 0.0001), LDH (Figure 3E, *p* < 0.0001), Cr (Figure 3F, *p* < 0.01), BUN (Figure 3G, *p* < 0.0001), and amylase (Figure 3H, *p* < 0.001) compared in the sedentary groups. Low-dose HMPA administration significantly decreased plasma BUN levels compared to in the C group following exercise (Figure 3G, *p* < 0.05). Neither exercise nor HMPA administration affected plasma uric acid levels (Figure 3H).

Energy metabolism substances were also investigated in this study. Exhaustive exercise significantly increased plasma triglyceride (TG) (Figure 3M, *p* < 0.001) and free fatty acids (FFAs) levels (Figure 3N, *p* < 0.0001), while simultaneously causing a significant decrease in low-density lipoprotein cholesterol (LDL) (Figure 3J, *p* < 0.0001), high-density lipoprotein cholesterol (HDL) (Figure 3K, *p* < 0.01), total cholesterol (Figure 3L, *p* < 0.0001), and glucose (Figure 3O, *p* < 0.0001) levels. However, none of these significant differences were observed with HMPA administration.

### 2.3. Effect of HMPA Administration on Glucose Metabolism in Gastrocnemius

**Figure 4:**
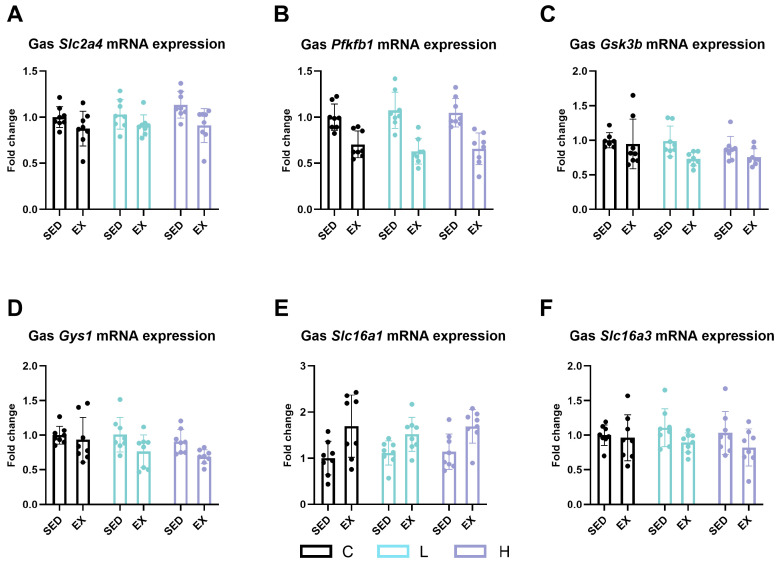
mRNA expression of (**A**) *Slc2a4* (*Glut4*), (**B**) *Pfkfb1*, (**C**) *Gsk3b*, (**D**) *Gys1*, (**E**) *Slc16a1* (*Mct1*), and (**F**) *Slc16a3* (*Mct4*) in gastrocnemius. Gas, gastrocnemius; *Slc2a4* (*Glut4*), solute carrier family 2 (facilitated glucose transporter), member 4; *Pfkfb1*, 6-phosphofructo-2-kinase/fructose-2, 6-biphosphatase 1; *Gsk3b*, glycogen synthase kinase 3 beta; *Gys1*, glycogen synthase 1; *Slc16a1* (*Mct1*), solute carrier family 16 (monocarboxylic acid transporters), member 1; *Slc16a3* (*Mct4*), solute carrier family 16 (monocarboxylic acid transporters), member 3; C group, vehicle control group; L group, low-dose HMPA administration group; H group, high-dose HMPA administration group; SED, sedentary; EX, exercise. Values are the means ± standard deviation (SD).

We measured gene expression levels related to glucose metabolism in the gastrocnemius, focusing on several key enzymes and transporters: solute carrier family 2 (facilitated glucose transporter), member 4 (*Slc2a4* or *Glut4*); 6-phosphofructo-2-kinase/fructose-2,6-biphosphatase 1 (*Pfkfb1*); glycogen synthase kinase 3 beta (*Gsk3b*); glycogen synthase 1 (*Gys1*); and solute carrier family 16, member 1 and member 3, commonly known as monocarboxylic acid transporter *(Mct*) 1 and *Mct4*, respectively. Our findings revealed that exercise significantly decreased the mRNA expression levels of *Slc2a4* (*Glut4*) (Figure 4A, *p* < 0.0001), *Pfkfb1* (Figure 4B, *p* < 0.0001), *Gsk3b* (Figure 4C, *p* < 0.05), *Gys1* (Figure 4D, *p* < 0.05), and *Mct4* (Figure 4F, *p* < 0.05). Conversely, the expression levels of *Mct1* significantly increased after exercise (Figure 4E, *p* < 0.0001). However, no significant differences were found after HMPA administration.

### 2.4. Effect of HMPA Administration on Glucose Metabolism in Liver

**Figure 5:**
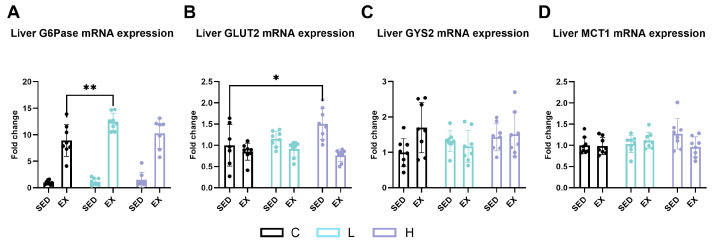
mRNA expression of (**A**) *G6pc1* (*G6Pase*), (**B**) *Slc2a2* (*Glut2*), (**C**) *Gys2*, and (**D**) *Slc16a1* (*Mct1*) in liver. *G6pc1* (*G6Pase*), glucose-6-phosphatase catalytic subunit 1; *Slc2a2* (*Glut2*), solute carrier family 2 (facilitated glucose transporter), member 2; *Gys2*, glycogen synthase 2; *Slc16a1* (*Mct1*), solute carrier family 16 (monocarboxylic acid transporters), member 1; C group, vehicle control group; L group, low-dose HMPA administration group; H group, high-dose HMPA administration group; SED, sedentary; EX, exercise. Values are the means ± standard deviation (SD); * *p* < 0.05 and ** *p* < 0.01, compared with the C group.

Glucose-6-phosphatase catalytic subunit 1 (*G6pc1* or *G6Pase*) (Figure 5A, *p* < 0.0001) and *Slc2a2* (*Glut2*) (Figure 5B, *p* < 0.0001), which are related to glucose metabolism in the liver, were significantly increased following exhaustive exercise. Compared to in the CE group, low-dose HMPA administration significantly enhanced hepatic *G6pc1* (*G6Pase*) gene expression after exercise (Figure 5A, *p* < 0.05). Additionally, a significant interaction was observed between HMPA administration and exercise in *Slc2a2* (*Glut2*) gene expression (Figure 5B, *p* < 0.05). Compared to in the C group, the H group showed significantly increased *Slc2a2* (*Glut2*) gene expression (Figure 5B, *p* < 0.05). However, no significant effect of HMPA administration or exercise was observed in the expression levels of *Gys2* (Figure 5C) or *Mct1* (Figure 5D).

### 2.5. Effect of HMPA Administration on Lipid Metabolism in Gastrocnemius

**Figure 6:**
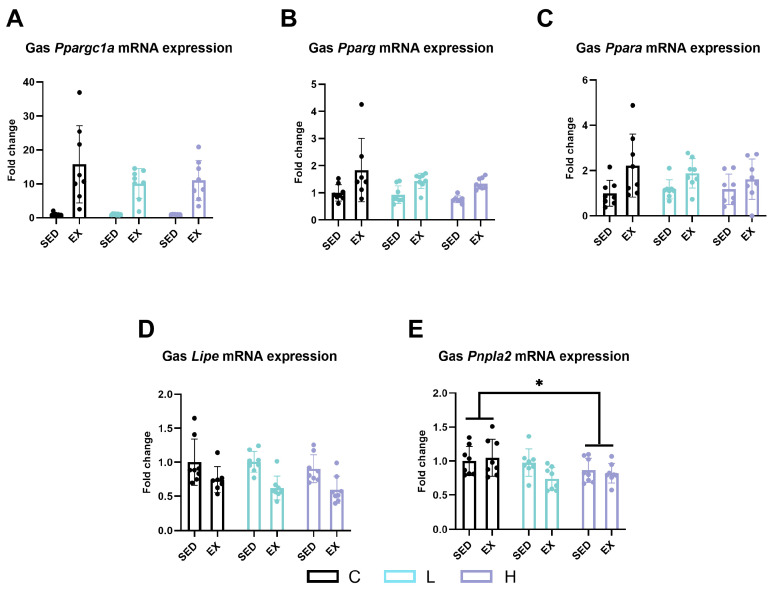
mRNA expression of (**A**) *Ppargc1a* (*Pgc-1alpha*), (**B**) *Pparg*, (**C**) *Ppara*, (**D**) *Lipe* (*HSL*), and (**E**) *Pnpla2* (*Atgl*) in gastrocnemius. *Ppargc1a* (*Pgc-1alpha*), peroxisome proliferative-activated receptor, gamma, coactivator 1 alpha; *Pparg*, peroxisome proliferator-activated receptor gamma; *Ppara*, peroxisome proliferator-activated receptor alpha; *Lipe* (*HSL*), hormone-sensitive lipase; *Pnpla2* (*Atgl*), patatin-like phospholipase domain containing 2; C group, vehicle control group; L group, low-dose HMPA administration group; H group, high-dose HMPA administration group; SED, sedentary; EX, exercise. Values are the means ± standard deviation (SD); * *p* < 0.05, compared with the C group.

To further explore the changes in lipid metabolism in the gastrocnemius, we evaluated the mRNA expression levels of peroxisome proliferative-activated receptor, gamma, coactivator 1 alpha (*Ppargc1a* or *Pgc-1alpha*), peroxisome proliferator-activated receptor gamma (*Pparg*), hormone-sensitive lipase (*Lipe* or *HSL*), patatin-like phospholipase domain containing 2 (*Pnpla2* or *Atgl*), and peroxisome proliferator-activated receptor alpha (*Ppara*), and found that exhaustive exercise significantly upregulated the expression levels of *Ppargc1a* (*Pgc-1alpha*) (Figure 6A, *p* < 0.0001), *Pparg* (Figure 6B, *p* < 0.001), and *Ppara* (Figure 6C, *p* < 0.01). The mRNA expression level of *Pnpla2* (*Atgl*) in high-dose administration mice was significantly lower than that in vehicle control mice (Figure 6E, *p* < 0.05).

### 2.6. Effect of HMPA Administration on Lipid Metabolism in Liver

**Figure 7:**
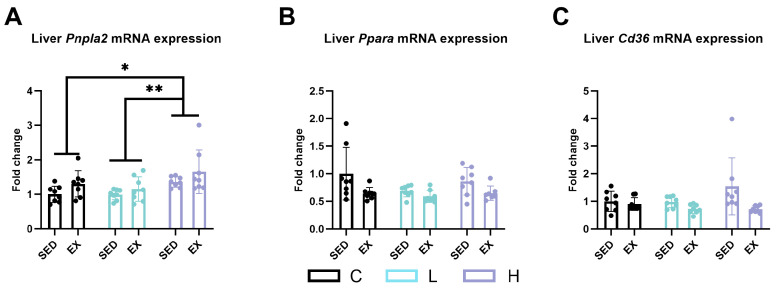
mRNA expression of (**A**) *Pnpla2* (*Atgl*), (**B**) *Ppara*, and (**C**) *Cd36* in liver. *Pnpla2* (*Atgl*), patatin-like phospholipase domain containing 2; *Ppara*, peroxisome proliferator-activated receptor alpha; *Cd36*, CD36 molecule; C group, vehicle control group; L group, low-dose HMPA administration group; H group, high-dose HMPA administration group; SED, sedentary; EX, exercise. Values are the means ± standard deviation (SD); * *p* < 0.05 and ** *p* < 0.01, compared with the C group.

*Pnpla2* (*Atgl*), *Ppara*, and CD36 molecule (*Cd36*) were measured to analyze the changes in liver lipid metabolism. Exhaustive exercise significantly reduced the expression levels of *Ppara* (Figure 7B, *p* < 0.01) and *Cd36* (Figure 7C, *p* < 0.01), while increasing the expression of *Pnpla2* (*Atgl*) (Figure 7A, *p* < 0.05). Compared to both the vehicle control group and the low-dose administration group, high-dose administration mice had significantly higher mRNA expression levels of *Pnpla2* (*Atgl*) (Figure 7A, *p* < 0.05).

### 2.7. Effect of HMPA Administration on Muscle Satellite Cell Proliferation and Differentiation

**Figure 8:**
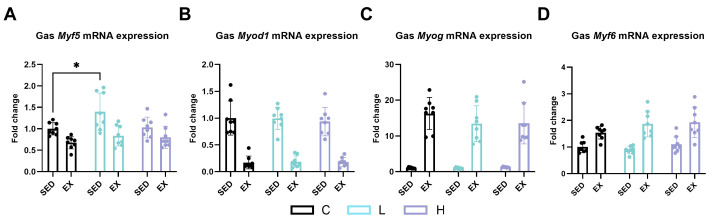
mRNA expression of (**A**) *Myf5*, (**B**) *Myod1*, (**C**) *Myog*, and (**D**) *Myf6* in gastrocnemius. Gas, gastrocnemius; *Myf5*, myogenic factor 5; *Myod1*, myoblast determination protein 1; *Myog*, myogenin; *Myf6*, myogenic factor 6; C group, vehicle control group; L group, low-dose HMPA administration group; H group, high-dose HMPA administration group; SED, sedentary; EX, exercise. Values are the means ± standard deviation (SD); * *p* < 0.05, compared with the C group.

To elucidate the underlying mechanisms contributing to the improvement in grip strength following HMPA administration, we analyzed the expression of genes related to myogenesis in the gastrocnemius muscle. Low-dose administration of HMPA significantly increased myogenic factor 5 (*Myf5*) mRNA expression compared to vehicle control mice (Figure 8A, *p* < 0.05). However, HMPA administration did not affect the mRNA levels of myoblast determination protein 1 (*Myod1*) (Figure 8B), myogenin (*Myog*) (Figure 8C), or myogenic factor 6 (*Myf6*) (Figure 8D).

### 2.8. Effect of HMPA Administration on Muscle Ubiquitin–Proteasome and Autophagic Lysosomal Pathways

**Figure 9:**
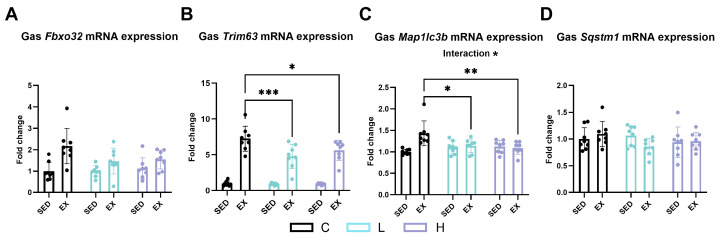
mRNA expression of (**A**) *Fbxo32* (*ATROGIN1*), (**B**) *Trim63* (*MuRF1*), (**C**) *Map1lc3b* (*LC3b*), and (**D**) *Sqstm1* (*p62*) in gastrocnemius. Gas, gastrocnemius; *Fbxo32* (*ATROGIN1*), F-box protein 32; *Trim63* (*MuRF1*), tripartite motif-containing 63; *Map1lc3b* (*LC3b*), microtubule-associated protein 1 light chain 3 beta; *Sqstm1* (*p62*), sequestosome 1; C group, vehicle control group; L group, low-dose HMPA administration group; H group, high-dose HMPA administration group; SED, sedentary; EX, exercise. Values are the means ± standard deviation (SD); * *p* < 0.05, ** *p* < 0.01, and *** *p* < 0.001, compared with the C group.

Exhaustive exercise increased the expression of F-box protein 32 (*Fbxo32* or *ATROGIN1*) (Figure 9A, *p* < 0.0001), tripartite motif-containing 63 (*Trim63* or *MuRF1*) (Figure 9B, *p* < 0.0001), and autophagy-related gene microtubule-associated proteins 1A/1B light chain 3B (*Map1lc3b* or *LC3b*) (Figure 9C, *p* < 0.01). HMPA administration, at both low and high doses, reduced the expression of *Trim63* (*MuRF1*) (Figure 9B) and *Map1lc3b* (*LC3b*) after exercise (Figure 9C). No significant differences were observed in the expression of sequestosome 1 (*Sqstm1* or *p62*) (Figure 9D) after exercise or HMPA administration.

## 3. Discussion

There is limited literature addressing the effect of HMPA on muscle function and its potential protective role against tissue damage induced by exhaustive exercise. In the present study, we evaluated the potential beneficial effects of HMPA administration on muscle function and tissue damage after a physical challenge in mice. The primary finding of our study indicates that 14-day HMPA administration improves grip strength, and low-dose HMPA reduces plasma BUN levels after exhaustive exercise. However, HMPA administration did not improve exercise endurance capacity in mice.

Facilitating energy production and substrate utilization is believed to be associated with optimizing muscle function. Preserving muscle glycogen and sustaining higher blood glucose levels are key factors in maintaining and improving exercise performance [27,28]. Swim training has been reported to improve grip strength by optimizing muscle energy metabolism in a muscle dysfunction model of mice [29]. The liver is the major metabolic organ. During exercise, it increases glucose production by activating gluconeogenesis and glycogenolysis, and modulates lipid metabolism in response to the increased energy demands [30]. Thus, we investigated the effects of HMPA on glucose and lipid metabolism, focusing on circulating substrates and mRNA expression levels in the liver and gastrocnemius. Our results showed that HMPA did not affect the plasma levels of energy substances in either sedentary or exercised mice. Regarding mRNA expression, high-dose HMPA increased the gene expression level of *Slc2a2* (*Glut2*) in sedentary mice compared to in the C group. Additionally, low-dose HMPA significantly increased the expression level of *G6pc1* (*G6Pase*) after exercise. *G6pc1* (*G6Pase*) catalyzes the final step in converting glycogen and other substrates to glucose to support the increased energy needs induced by prolonged exercise [31]. Our results suggest that HMPA could improve glucose metabolism and glucose utilization in the liver, potentially enhancing glucose supply during exercise. Despite this, these beneficial effects were not observed in the plasma glucose levels. During prolonged exercise, FFAs serve as a primary energy source, which can be taken up from the circulation and originate from the lipolysis of triacylglycerol [32]. *Pnpla2* (*Atgl*) is a crucial enzyme involved in the hydrolysis of triglycerides in adipocytes and other cells, playing an essential role in supplying FFAs during exercise [33]. We observed that high-dose HMPA enhanced *Pnpla2* (*Atgl)* expression level in the liver but suppressed it in the gastrocnemius during exercise, indicating that high-dose HMPA may promote the release and use of FFAs from liver triglycerides while conserving muscle triglycerides. This dual action may tend to delay the depletion of muscular energy, thereby potentially improving exercise performance. However, these findings did not adequately explain the mechanism by which HMPA administration enhances grip strength in sedentary mice.

MRFs, comprising *Myod1*, *Myf5*, *Myog*, and *Myf6*, are master transcription factors that are upregulated during myogenesis and play an important role in influencing stem cells to differentiate into myogenic lineage cells. *Myf5* and *Myod1* are essential for the early stage of myogenesis, primarily involved in the determination and specification of myoblasts [34,35,36]. *Myog* and *Myf6* mediate the late stages of myogenesis, promoting the formation and maturation of myotubes [35,37]. Numerous studies have confirmed that dietary polyphenolic compounds can affect satellite cells and myoblast proliferation and differentiation [19,38,39,40]. HMCA has been reported to promote hypertrophic growth of fast skeletal muscle by upregulating relative mRNA expression levels of MRFs, specifically *Myod1* and *Myog* [19]. Another well-known polyphenolic compound, catechins, has been reported to stimulate satellite cell activation by inducing *Myf5* expression, which contributes to muscle regeneration after muscle damage [41]. *Myf5* expression is important for muscle formation and growth. In this study, we investigated the effect of HMPA administration on the regulation of MRFs expression. We found that low-dose HMPA administration significantly increased the expression of *Myf5* in sedentary mice, indicating HMPA may enhance muscle development or regeneration processes through the upregulation of myogenic factors. These results suggest that HMPA could be used as a supplement ingredient to improve muscle function in sedentary or muscle-wasting conditions. However, it should be noted that improving muscle strength can result from the interaction of multiple regulatory factors. The increase in *Myf5* induced by low-dose HMPA may be just one of the potential contributors. Further studies are needed to investigate additional potential mechanisms, such as assessing muscle mass and the pathways regulating protein synthesis in muscle.

Protein catabolism serves as a subsidiary energy substrate during prolonged endurance exercise when primary energy stores, carbohydrates and fat, are insufficient or depleted. BUN is a waste byproduct from the breakdown of proteins and amino acids, which is excreted by the kidneys [42]. Low-dose HMPA administration significantly reduced the increased BUN levels caused by exhaustive exercise. Our findings indicate that HMPA may provide a protective effect against muscle protein degradation caused by exhaustive exercise. We speculated that this protective effect of HMPA may be attributed to its modulation of signaling pathways of muscle protein degradation, specifically the ubiquitin-proteasome and autophagic-lysosomal pathways. In the present study, our findings demonstrate that exhaustive exercise leads to an increase in muscle protein degradation, as indicated by elevated mRNA expression levels of the muscle atrophy markers *Fbxo32* (*ATROGIN1*), *Trim63* (*MuRF1*), and the autophagy-related protein *Map1lc3b* (*LC3b*). These findings are consistent with the results of Zhang et al. [43]. Considerable evidence shows that utilizing nutritional supplements to inhibit muscle protein degradation following intense resistance or endurance exercise can optimize protein synthesis, repair muscle damage, and stimulate training adaptations [44,45]. According to a previous study involving a mouse model of diabetes-induced muscle atrophy, HMCA was demonstrated to increase myofiber size and enhance grip strength. These improvements are attributed to its inhibitory effects on the expression of FBXO-32 and MURF-1 in skeletal muscle, thereby reducing muscle degradation and improving muscle function [46]. We observed that HMPA inhibited the expression of *Trim63* (*MuRF1*) and *Map1lc3b* (*LC3b*). These results suggest that HMPA could prevent muscle protein degradation by ameliorating the ubiquitin–proteasome and the autophagic lysosomal pathways during exhaustive exercise. This mechanism may be beneficial for preserving muscle mass and facilitating subsequent muscle repair. However, these findings are confined to the time point at which muscle tissue was collected immediately after exercise. The time-course changes after exercise have not been determined. Future research needs to elucidate more details about the effects of HMPA on muscle repair, regeneration, and adaptation.

In this study, HMPA administration did not exhibit a dose-dependent effect on improving muscle function. The dosages used in this study were selected based on previous research on HMCA, the parent compound of HMPA, which showed dose-dependent effects on improving muscle function in animal models [18,47]. However, our findings revealed that low-dose HMPA demonstrated greater benefits, such as increased grip strength and decreased BUN levels after exercise, underscoring its potent bioactivity. Conversely, high-dose HMPA did not show these beneficial effects, which may be attributed to metabolic saturation or negative feedback mechanisms induced by consecutive high-dose administration, potentially leading to diminished muscle responsiveness.

Despite these interesting findings, our study has several limitations. First, the sample size was relatively small, which may have limited the power to detect more subtle differences between treatment groups. Second, while HMPA is known to possess antioxidant properties [12,15], the relationship between its antioxidant effects and muscle development remains unexplored. Finally, as this is the first study to investigate the effects of HMPA on muscle strength and exercise endurance capacity in mice, further research is warranted to determine the optimal dosage and duration of HMPA treatment for improving muscle health.

In summary, our findings provide initial evidence for the potential of HMPA as a dietary supplement to enhance muscle function and reduce protein catabolism induced by exhaustive exercise. However, more comprehensive studies are needed to understand its mechanisms of action and establish the most effective administration protocols.

## 4. Materials and Methods

### 4.1. Animals

Eight-week-old male C57BL/6 mice (*n* = 48) were obtained from Takasugi Experimental Animals Supply Co., Ltd. (Kasukabe, Japan). Three or four mice were housed together in plastic cages under a suitable growth environment with automatic temperature and relative humidity controls, along with a 12/12 h light/dark cycle. Mice were given CE-2 standard pelleted chow and allowed free access to water. The care of mice adhered to the Guiding Principles for the Care and Use of Animals laid out by the Waseda University Institutional Animal Care and Use Committee (approved number: A23-127).

### 4.2. HMPA Administration

A formulation containing 25.4% HMPA and dextrin, used in this study, was provided by Maruzen Pharmaceuticals Co., Ltd. (Hiroshima, Japan). Since the optimal dosage and duration for HMPA treatment have not been well researched, we referenced studies on HMCA and muscle function due to their similar metabolic benefits and characteristics [16,17,18,19,20,21,48]. Because of the low toxicity and rapid metabolism of HMPA, we selected two significantly different doses: 50 mg/kg and 500 mg/kg (corresponding to actual HMPA dosages of 12.7 mg/kg and 127 mg/kg, respectively), which were confirmed to be non-toxic to mice in our preliminary study.

Following a one-week acclimatization period, mice were stratified into three experimental cohorts based on their average body weight and baseline grip strength measurements, ensuring equitable distribution. These groups comprised a vehicle control group (distilled water, 10 mL/kg), a low-dose HMPA group (50 mg/kg body weight (BW)/day), and a high-dose HMPA group (500 mg/kg BW/day). Each freshly prepared solution or distilled water was then orally administered to mice through a plastic gastric tube for 14 consecutive days. The administration was performed at approximately the same time of day (between 9:30 and 11:30 a.m.) to limit potential influences.

### 4.3. Functional Assessment of Skeletal Muscle

**Figure 10:**
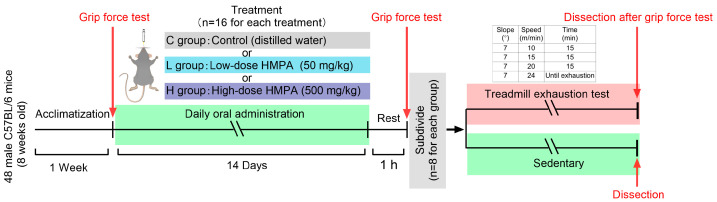
Schematic of the experimental protocol.

Grip force test and treadmill exhaustion test were conducted to evaluate the functional capacity of the skeletal muscle. The experimental protocol is depicted in Figure 10.

A grip-strength meter (GPM-101BV-C, Melquest, Toyama, Japan) was employed to assess muscle force at three time points: before oral administration, one hour after the final oral administration, and immediately after exercise (for exercise groups only) to evaluate muscle fatigue. The protocol has been described previously [49]. In brief, the mouse was allowed to grip a mesh bar with its forelimbs and hind limbs, and then the tail of the mouse was pulled backward at a constant speed until the forelimbs released. This measurement was conducted three consecutive times by the same operator blinded to the treatments. The maximum force value of the three trials was defined as absolute grip strength, recorded in grams. Relative grip strength was calculated as the absolute grip strength divided by body weight. The absolute and relative grip strength were used to analyze the grip strength.

The treadmill exhaustion test was performed to assess endurance capacity of the mice. All mice were allowed to acclimate to a motorized treadmill (Kyoto, Japan) two days prior, by running for 10 min at a speed of 15 m/min with no incline. On the testing day, each of the vehicle control group, low-dose HMPA group, and high-dose HMPA group was subdivided into exercise (EX) and sedentary (SED) groups: (1) C group (vehicle control administration + sedentary group, n = 8), (2) CE group (vehicle control administration + exercise group, n = 8), (3) L group (low-dose HMPA administration + sedentary group, n = 8), (4) LE group (low-dose HMPA administration + exercise group, n = 8), (5) H group (high-dose HMPA administration + sedentary group, n = 8), (6) HE group (high-dose HMPA administration + exercise group, n = 8). After one hour of the final oral administration, the exercise subgroups underwent exhaustive exercise beginning at a 7° incline and 10 m/min for 15 min, subsequently increasing by 5 m/min every 15 min for the next 30 min, and finally increasing to 24 m/min until the mice met the criteria for exhaustion: mice failed to maintain the speed and frequently turned around despite the persistent stimulation by gently brushing the back and tail of the mice. An operator who was blinded to the group assignment was responsible for the judgment of exhaustion and recorded the time of exhaustion. Immediately after exhaustion, the mice were subjected to the final grip force test and then anesthetized by isoflurane inhalation (Abbott, Tokyo, Japan). Blood samples were collected from the inferior vena cava into heparin-coated tubes to obtain plasma. Following euthanasia of the mice by cervical vertebra dislocation, the liver and gastrocnemius were harvested and immediately frozen in liquid nitrogen. All samples were stored at −80 °C until analyses.

### 4.4. Assessment of Plasma Biochemical Markers

Tissue damage and metabolic markers, including liver function (AST, ALT, and albumin levels), renal function (BUN, Cr, and uric acid levels), pancreatic enzymes (amylase), muscle damage markers (LDH and CK levels), lipid profiles (TG, FFA, total cholesterol, LDL, and HDL levels), and blood glucose level were measured by Kotobiken Medical Laboratories (Tokyo, Japan).

### 4.5. Quantitative RT-PCR

Total RNA was extracted using the NucleoSpin RNA Kit (Takara, Kusatsu, Japan) according to the protocol provided by the manufacturer. The concentration and purity of the RNA samples were evaluated with the NanoDrop ND-1000 spectrometer (NanoDrop Technologies, Wilmington, DE, USA). cDNA was synthesized using the High-Capacity cDNA Reverse Transcription Kit (Applied Biosystems, Foster City, CA, USA). PCR amplification was conducted with the Applied Biosystems QuantStudio 12K Flex Real-Time PCR System (Thermo Fisher Scientific, Foster City, CA, USA) using Fast SYBR™ Green Master Mix (Applied Biosystems, Foster City, CA, USA) in adherence to the recommended protocol. Ribosomal protein S18 (*rps18*) served as a housekeeping gene, and the relative expression levels of the target genes were determined using the 2^–∆∆Ct^ method. The primer sequences for the PCR reactions for each target gene are detailed in Table 1.

### 4.6. Statistical Analysis

GraphPad Prism 10 (GraphPad Software, La Jolla, CA, USA) was applied for statistical analysis. All data were shown as the mean ± standard deviation (SD). In cases of extreme data, the interquartile range (IQR) method was used to identify and exclude outliers. The grip force of mice after administration and exhaustion time were analyzed by one-way ANOVA followed by Tukey’s post hoc test. Student’s *t*-test was applied to compare the difference between the C group and the HMPA-administered (L + H) group. Additionally, a two-way ANOVA was applied to investigate the effect of HMPA administration and time, as well as the effect of HMPA administration and/or exercise. If a significant interaction was identified, simple main-effects analysis and Tukey’s post hoc test were applied. When main effects were identified as statistically significant, main-effects analysis and Tukey’s post hoc test were applied. *p* < 0.05 was considered statistically significant.

## 5. Conclusions

This study demonstrated that HMPA enhances grip strength after 14 days of administration. Low-dose HMPA may promote muscle proliferation and differentiation by upregulating *Myf5* expression. Furthermore, our results suggest that low-dose HMPA reduces BUN levels after exhaustive exercise, indicating an important role for HMPA in preserving muscle protein during exhaustive exercise. This protective effect may be attributed to the inhibition of ubiquitin-proteasome and autophagic lysosomal pathways during exhaustive exercise. Although HMPA increased mRNA expression levels related to glucose and lipid metabolism in the liver, these changes did not affect plasma levels of metabolites or endurance performance in mice. These findings suggest potential applications of HMPA as a natural dietary supplement to improve muscle development and preserve muscle mass.

## Figures and Tables

**Table 1:** Primer sequence for Real-Time PCR analysis.

Gene	Forward	Reverse
*Slc2a4* (*Glut4*)	GCTGGTGTGGTCAATACGGTCT	GCAGAGCCACGGTCATCAAGAT
*Slc2a2* (*Glut2*)	CCTCATCATTGCTGGACGAAGTGT	CCTGAGTGTGGTTGGAGCGATCT
*Slc16a1* (*Mct1*)	TCAGTGCAACGACCAGTGAAGT	CCGCAACCAGACAGACAACCA
*Slc16a3* (*Mct4*)	ACGGGTTTCTCCTACGCCTTCC	AGAGCGGTCCTGTGCCATAGAG
*Pfkfb1*	GCGGTGCCTTCTAGCATACTTCC	TCACAGCCTCCACATTCAGGTAGA
*Gsk3b*	TGGTGTGGATCAGTTGGTGGAA	TCCTGCTCCTGGTGAGTCCTT
*Gys1*	CTGTCCTGTTCGGCTTCCTCAC	GGCAACCACATACGGCTTCTCT
*Gys2*	GCTTCCGCTCTCCAGACGATTC	TGCCCAGGTATCTCCAGTCCAG
*G6pc1* (*G6Pase*)	GTGGCAGTGGTCGGAGACT	ACGGGCGTTGTCCAAAC
*Ppargc1a* (*Pgc-1alpha)*	ACACAACCGCAGTCGCAACAT	GCAGTTCCAGAGAGTTCCACACTT
*Ppara*	GCGGGAAAGACCAGCAACAAC	CAGCAGTGGAAGAATCGGACCT
*Pparg*	GATGGAAGACCACTCGCATT	AACCATTGGGTCAGCTCTTG
*Lipe* (*HSL*)	GGAAGGACAGGACAG-CAAGGTACT	CGCCTCCGTGGATGTGAACAAC
*Pnpla2* (*Atgl*)	TGTCCTTCACCATCCGCTTGTT	TGCTACCCGTCTGCTCTTTCAT
*Cd36*	TGCGACATGATTAATGGCACAGAC	TCCGAACACAGCGTAGATAGACCT
*Myf5*	CTCAGGAATGCCATCCGCTACA	CCGTCAGAGCAGTTGGAGGT
*Myod1*	CGCAACGCCATCCGCTACAT	GCATCTGAGTCGCCACTGTAGT
*Myog*	GCAGGCTCAAGAAAGTGAATGA	TAGGCGCTCAATGTACTGGAT
*Myf6*	CCTCAGCCTCCAGCAGTCTTCA	TTACTTCTCCACCACCTCCTCCAC
*Trim63* (*MuRF1*)	CGACATCTTCCAGGCTGCGAAT	ATCACTTCATGGCGGCACGAG
*Fbxo32* (*ATROGIN1*)	GCTGGATTGGAAGAAGATG	AGAGAATGTGGCAGTGTT
*Map1lc3b* (*LC3b*)	CCAAGCCTTCTTCCTCCT	CTCTCACTCTCGTACACTTC
*Sqstm1 (p62)*	CAGGCACAGAAGACAAGAG	CCGACTCCAAGGCTATCT
rps18	TTCTGGCCAACGGTCTAGACAAC	CCAGTGGTCTTGGTGTGCTGA

*Slc2a4* (*Glut4*), solute carrier family 2 (facilitated glucose transporter), member 4; *Slc2a2* (*Glut2*), solute carrier family 2 (facilitated glucose transporter), member 2; *Slc16a1* (*Mct1*), solute carrier family 16 (monocarboxylic acid transporters), member 1; *Slc16a3* (*Mct4*), solute carrier family 16 (monocarboxylic acid transporters), member 3; *Pfkfb1*, 6-phosphofructo-2-kinase/fructose-2, 6-biphosphatase 1; *Gsk3b*, glycogen synthase kinase 3 beta; *Gys1*, glycogen synthase 1; *Gys2*, glycogen synthase 2; *G6pc1* (*G6Pase*), glucose-6-phosphatase catalytic subunit 1; *Ppargc1a* (*Pgc-1alpha*), peroxisome proliferative-activated receptor, gamma, coactivator 1 alpha; *Ppara*, peroxisome proliferator-activated receptor alpha; *Pparg*, peroxisome proliferator-activated receptor gamma; *Lipe* (*HSL*), hormone-sensitive lipase; *Pnpla2* (*Atgl*), patatin-like phospholipase domain containing 2; *Cd36*, CD36 molecule; *Myf5*, myogenic factor 5; *Myod1*, myoblast determination protein 1; *Myog*, myogenin; *Myf6*, myogenic factor 6; *Fbxo32* (*ATROGIN1*), F-box protein 32; *Trim63* (*MuRF1*), tripartite motif-containing 63; *Map1lc3b* (*LC3b*), microtubule-associated protein 1 light chain 3 beta; *Sqstm1* (*p62*), sequestosome 1; rps18, ribosomal protein S18.

## Data Availability

Data are contained within this article or Supplementary Materials.

## Supplementary Materials

The following supporting information can be downloaded at: https://www.mdpi.com/article/10.3390/ijms25126627/s1.

## References

[1] Periasamy M., Herrera J.L., Reis F. (2024). Skeletal Muscle Thermogenesis and Its Role in Whole Body Energy Metabolism. Diabetes Metab. J..

[2] Argilés J.M., Campos N., Lopez-Pedrosa J.M., Rueda R., Rodriguez-Mañas L. (2016). Skeletal Muscle Regulates Metabolism via Interorgan Crosstalk: Roles in Health and Disease. J. Am. Med. Dir. Assoc..

[3] Pinto A.J., Bergouignan A., Dempsey P.C., Roschel H., Owen N., Gualano B., Dunstan D.W. (2023). Physiology of Sedentary Behavior. Physiol. Rev..

[4] Rezuş E., Burlui A., Cardoneanu A., Rezuş C., Codreanu C., Pârvu M., Rusu-Zota G., Tamba B.I. (2020). Inactivity and Skeletal Muscle Metabolism: A Vicious Cycle in Old Age. Int. J. Mol. Sci..

[5] Tipton K.D., Ferrando A.A. (2008). Improving Muscle Mass: Response of Muscle Metabolism to Exercise, Nutrition and Anabolic Agents. Essays Biochem..

[6] McGee S.L., Hargreaves M. (2020). Exercise Adaptations: Molecular Mechanisms and Potential Targets for Therapeutic Benefit. Nat. Rev. Endocrinol..

[7] Zammit P.S. (2017). Function of the Myogenic Regulatory Factors Myf5, MyoD, Myogenin and MRF4 in Skeletal Muscle, Satellite Cells and Regenerative Myogenesis. Semin. Cell Dev. Biol..

[8] Li J., Li Y., Atakan M.M., Kuang J., Hu Y., Bishop D.J., Yan X. (2020). The Molecular Adaptive Responses of Skeletal Muscle to High-Intensity Exercise/Training and Hypoxia. Antioxidants.

[9] Li Q., Yang H., Song S., Liu J., Wang Z., Wang J. (2022). Bioactive Components in Whole Grains for the Regulation of Skeletal Muscle Function. Foods.

[10] Wang L., Xu Z., Ling D., Li J., Wang Y., Shan T. (2022). The Regulatory Role of Dietary Factors in Skeletal Muscle Development, Regeneration and Function. Crit. Rev. Food Sci. Nutr..

[11] Tian Y., Xia T., Qiang X., Zhao Y., Li S., Wang Y., Zheng Y., Yu J., Wang J., Wang M. (2022). Nutrition, Bioactive Components, and Hepatoprotective Activity of Fruit Vinegar Produced from Ningxia Wolfberry. Molecules.

[12] Lee S.H., Yeo D., Hong J. (2020). Effect of Dihydroferulic Acid Obtained from Fermented Rice Bran Extract on Neuroprotection and Behavioral Recovery in an Ischemic Rat Model. Food Sci. Technol..

[13] Ohue-Kitano R., Taira S., Watanabe K., Masujima Y., Kuboshima T., Miyamoto J., Nishitani Y., Kawakami H., Kuwahara H., Kimura I. (2019). 3-(4-Hydroxy-3-methoxyphenyl)propionic Acid Produced from 4-Hydroxy-3-methoxycinnamic Acid by Gut Microbiota Improves Host Metabolic Condition in Diet-Induced Obese Mice. Nutrients.

[14] Rechner A.R., Spencer J.P., Kuhnle G., Hahn U., Rice-Evans C.A. (2001). Novel Biomarkers of the Metabolism of Caffeic Acid Derivatives in Vivo. Free Radic. Biol. Med..

[15] Shimoji Y., Tamura Y., Nakamura Y., Nanda K., Nishidai S., Nishikawa Y., Ishihara N., Uenakai K., Ohigashi H. (2002). Isolation and Identification of DPPH Radical Scavenging Compounds in Kurosu (Japanese Unpolished Rice Vinegar). J. Agric. Food Chem..

[16] Chowdhury S., Ghosh S., Das A.K., Sil P.C. (2019). Ferulic Acid Protects Hyperglycemia-Induced Kidney Damage by Regulating Oxidative Insult, Inflammation and Autophagy. Front. Pharmacol..

[17] Zhang S., Wang P., Zhao P., Wang D., Zhang Y., Wang J., Chen L., Guo W., Gao H., Jiao Y. (2018). Pretreatment of Ferulic Acid Attenuates Inflammation and Oxidative Stress in a Rat Model of Lipopolysaccharide-Induced Acute Respiratory Distress Syndrome. Int. J. Immunopathol. Pharmacol..

[18] Wang Y., Chen X., Huang Z., Chen D., Yu B., Chen H., Yu J., Luo Y., Zheng P., He J. (2021). Effects of Dietary Ferulic Acid Supplementation on Growth Performance and Skeletal Muscle Fiber Type Conversion in Weaned Piglets. J. Sci. Food Agric..

[19] Wen Y., Ushio H. (2017). Ferulic Acid Promotes Hypertrophic Growth of Fast Skeletal Muscle in Zebrafish Model. Nutrients.

[20] You Y., Park J., Yoon H.G., Lee Y.H., Hwang K., Lee J., Kim K., Lee K.W., Shim S., Jun W. (2009). Stimulatory Effects of Ferulic Acid on Endurance Exercise Capacity in Mice. Biosci. Biotechnol. Biochem..

[21] You Y., Kim K., Yoon H.G., Lee K.W., Lee J., Chun J., Shin D.H., Park J., Jun W. (2010). Chronic Effect of Ferulic Acid from Pseudosasa Japonica Leaves on Enhancing Exercise Activity in Mice. Phytother Res..

[22] Vashisth P., Kumar N., Sharma M., Pruthi V. (2015). Biomedical Applications of Ferulic Acid Encapsulated Electrospun Nanofibers. Biotechnol. Rep. (Amst)..

[23] Rezaei A., Varshosaz J., Fesharaki M., Farhang A., Jafari S.M. (2019). Improving the Solubility and in Vitro Cytotoxicity (Anticancer Activity) of Ferulic Acid by Loading it into Cyclodextrin Nanosponges. Int. J. Nanomed..

[24] Li D., Rui Y.X., Guo S.D., Luan F., Liu R., Zeng N. (2021). Ferulic Acid: A Review of Its Pharmacology, Pharmacokinetics and Derivatives. Life Sci..

[25] Baeza G., Bachmair E.M., Wood S., Mateos R., Bravo L., de Roos B. (2017). The Colonic Metabolites Dihydrocaffeic Acid and Dihydroferulic Acid Are More Effective Inhibitors of in Vitro Platelet Activation Than Their Phenolic Precursors. Food Funct..

[26] Ohue-Kitano R., Masujima Y., Nishikawa S., Iwasa M., Nishitani Y., Kawakami H., Kuwahara H., Kimura I. (2023). 3-(4-Hydroxy-3-methoxyphenyl)propionic Acid Contributes to Improved Hepatic Lipid Metabolism Via GPR41. Sci. Rep..

[27] Inamura N., Taniguchi H., Yoshida S., Nishioka M., Ishihara K. (2024). A Comparative Observational Study of Carbohydrate Intake and Continuous Blood Glucose Levels in Relation to Performance in Ultramarathon. Sci. Rep..

[28] Noakes T.D. (2022). What Is the Evidence That Dietary Macronutrient Composition Influences Exercise Performance? A Narrative Review. Nutrients.

[29] Flis D.J., Dzik K., Kaczor J.J., Cieminski K., Halon-Golabek M., Antosiewicz J., Wieckowski M.R., Ziolkowski W. (2019). Swim Training Modulates Mouse Skeletal Muscle Energy Metabolism and Ameliorates Reduction in Grip Strength in a Mouse Model of Amyotrophic Lateral Sclerosis. Int. J. Mol. Sci..

[30] Rui L. (2014). Energy Metabolism in the Liver. Compr. Physiol..

[31] Zhang X., Yang S., Chen J., Su Z. (2019). Unraveling the Regulation of Hepatic Gluconeogenesis. Front. Endocrinol. (Lausanne).

[32] van Hall G. (2015). The Physiological Regulation of Skeletal Muscle Fatty Acid Supply and Oxidation During Moderate-Intensity Exercise. Sports Med..

[33] Dubé J.J., Sitnick M.T., Schoiswohl G., Wills R.C., Basantani M.K., Cai L., Pulinilkunnil T., Kershaw E.E. (2015). Adipose Triglyceride Lipase Deletion from Adipocytes, but Not Skeletal Myocytes, Impairs Acute Exercise Performance in Mice. Am. J. Physiol. Endocrinol. Metab..

[34] Wood W., Etemad S., Yamamoto M., Goldhamer D. (2015). MyoD-Expressing Progenitors Are Essential for Skeletal Myogenesis and Satellite Cell Development. Dev. Biol..

[35] Shirakawa T., Toyono T., Inoue A., Matsubara T., Kawamoto T., Kokabu S. (2022). Factors Regulating or Regulated by Myogenic Regulatory Factors in Skeletal Muscle Stem Cells. Cells.

[36] Yamamoto M., Legendre N.P., Biswas A.A., Lawton A., Yamamoto S., Tajbakhsh S., Kardon G., Goldhamer D.J. (2018). Loss of MyoD and Myf5 in Skeletal Muscle Stem Cells Results in Altered Myogenic Programming and Failed Regeneration. Stem Cell Rep..

[37] Ganassi M., Badodi S., Wanders K., Zammit P.S., Hughes S.M. (2020). Myogenin Is an Essential Regulator of Adult Myofibre Growth and Muscle Stem Cell Homeostasis. eLife.

[38] Kim A.R., Kim K.M., Byun M.R., Hwang J.H., Park J.I., Oh H.T., Jeong M.G., Hwang E.S., Hong J.H. (2017). (-)-Epigallocatechin-3-gallate Stimulates Myogenic Differentiation Through TAZ Activation. Biochem. Biophys. Res. Commun..

[39] Dugdale H.F., Hughes D.C., Allan R., Deane C.S., Coxon C.R., Morton J.P., Stewart C.E., Sharples A.P. (2018). The Role of Resveratrol on Skeletal Muscle Cell Differentiation and Myotube Hypertrophy During Glucose Restriction. Mol. Cell. Biochem..

[40] Jang Y.J., Son H.J., Choi Y.M., Ahn J., Jung C.H., Ha T.Y. (2017). Apigenin Enhances Skeletal Muscle Hypertrophy and Myoblast Differentiation by Regulating Prmt7. Oncotarget.

[41] Kim A.R., Kim K.M., Byun M.R., Hwang J.H., Park J.I., Oh H.T., Kim H.K., Jeong M.G., Hwang E.S., Hong J.H. (2017). Catechins Activate Muscle Stem Cells by Myf5 Induction and Stimulate Muscle Regeneration. Biochem. Biophys. Res. Commun..

[42] Lee E.C., Fragala M.S., Kavouras S.A., Queen R.M., Pryor J.L., Casa D.J. (2017). Biomarkers in Sports and Exercise: Tracking Health, Performance, and Recovery in Athletes. J. Strength Cond. Res..

[43] Zhang Q., Zheng J., Qiu J., Wu X., Xu Y., Shen W., Sun M. (2017). ALDH2 Restores Exhaustive Exercise-Induced Mitochondrial Dysfunction in Skeletal Muscle. Biochem. Biophys. Res. Commun..

[44] Liu S., Liu H., Yang L., Wang K., Chen N., Zhang T., Luo J. (2022). A Review of Rehabilitation Benefits of Exercise Training Combined with Nutrition Supplement for Improving Protein Synthesis and Skeletal Muscle Strength in Patients With Cerebral Stroke. Nutrients.

[45] Guimarães-Ferreira L., Cholewa J.M., Naimo M.A., Zhi X.I., Magagnin D., de Sá R.B., Streck E.L., Teixeira Tda S., Zanchi N.E. (2014). Synergistic Effects of Resistance Training and Protein Intake: Practical Aspects. Nutrition.

[46] Liu X., Liu M. (2023). Roles of Ferulic Acid on Muscle Atrophy and Grip Strength in Diabetic Mice. Pak. J. Pharm. Sci..

[47] Liu X., Liu M. (2023). Anti-Fatigue Effect of Ferulic Acid in Exercise Trained Mice. Acta Pol. Pharm.-Drug Res..

[48] Huang T., Li H., Chen X., Chen D., Yu B., He J., Luo Y., Yan H., Zheng P., Yu J. (2024). Dietary Ferulic Acid Increases Endurance Capacity by Promoting Skeletal Muscle Oxidative Phenotype, Mitochondrial Function, and Antioxidant Capacity. Mol. Nutr. Food Res..

[49] Ma S., Yang J., Tominaga T., Liu C., Suzuki K. (2021). A Low-Carbohydrate Ketogenic Diet and Treadmill Training Enhanced Fatty Acid Oxidation Capacity but Did Not Enhance Maximal Exercise Capacity in Mice. Nutrients.

