# Supplementary figures and images for: Effects of 3-(4-Hydroxy-3-methoxyphenyl)propionic Acid on Enhancing Grip Strength and Inhibiting Protein Catabolism Induced by Exhaustive Exercise
Source: Int J Mol Sci. 2024 Jun 16;25(12):6627. doi: 10.3390/ijms25126627 (PMC11203939; doi:10.3390/ijms25126627)

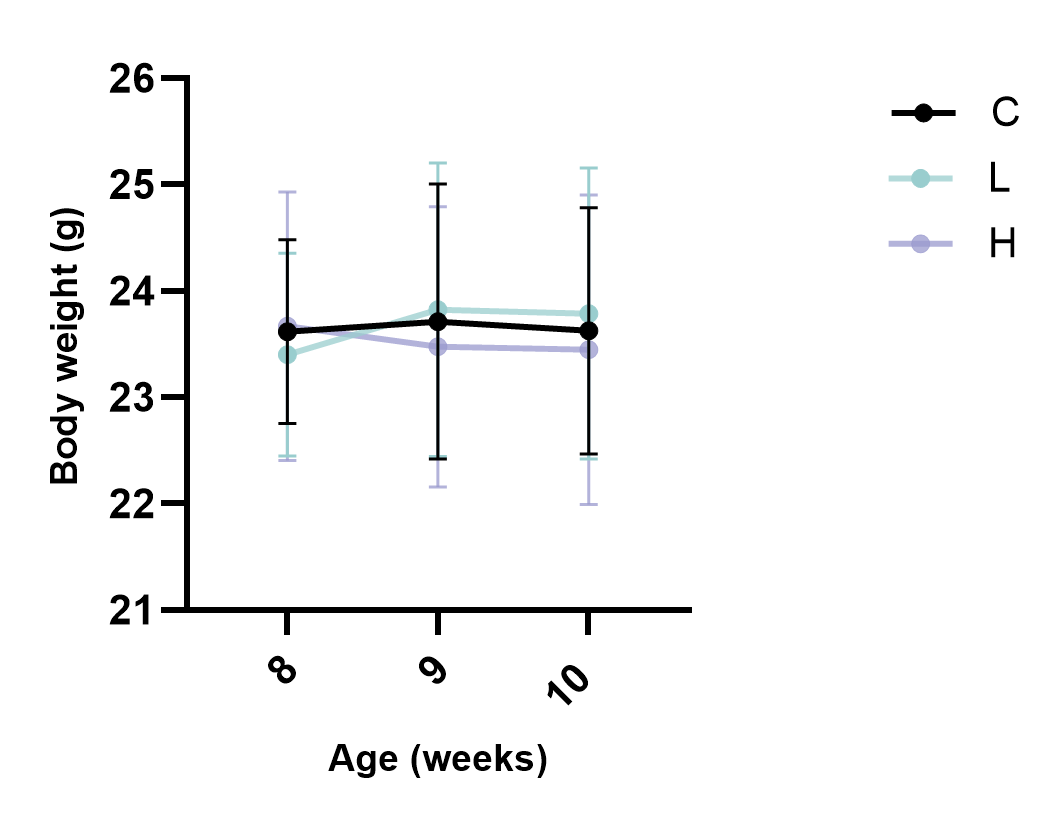

Supplement: Supplementary file 1 [file ijms-25-06627-s001.zip › Figure S1 Body-weight changes in mice receiving the different administration.tif]
